# Countermanding saccades in children

**DOI:** 10.3758/s13414-026-03342-4

**Published:** 2026-10-29

**Authors:** Timothy L. Hodgson, Jonathan Waddington, Kirsten Williams, Kun Guo

**Affiliations:** 1 School of Psychology, Sports Science and Wellbeing, College of Health and Science, University of Lincoln, Lincoln, LN6 7TS UK; 2 School of Health, Wellbeing and Social Sciences, Plymouth Marjon University, Plymouth, PL6 8BH UK

**Keywords:** Inhibitory control, Developmental neuroscience, Eye movement, Sticky fixation, Attention, Reaction times

## Abstract

**Abstract:**

The countermanding saccade task is a widely used test of reactive inhibitory oculomotor control. The test comprises “Go” trials, in which saccades are directed towards target onsets, interspersed with “Stop” trials, where a signal sometime after target onset indicates that a saccade should be withheld or “countermanded”. Computational models of the task assume a race to threshold between internal go and stop processes, the outcome of which determines whether a programmed response is cancelled. In this study, 181 typically developing primary-school-aged children completed child-friendly versions of the task. Three experiments examined the effect of task instruction (either to countermand saccades or ignore stop signals), stop signal characteristics (with and without an overlapping fixation cue) and practice (one versus two blocks). Children often failed to make saccades even when they were instructed to ignore the signal and make a response to the target on every trial. Saccade reaction times were longer on Stop compared with correct Go trials and significant “dips” (reductions in expected response frequency within discrete time bins) were observed in reaction time distributions associated with the change in the colour of the fixation stimulus that served as a stop signal. It is concluded that visual stop signals exert an exogenous inhibitory effect on saccades which decreases with increasing age. As such, care should be taken in interpreting the oculomotor stop signal task as a straightforward test of developing inhibitory control or the maturation of an internal stop process.

**Open access statement:**

Experiment task code, raw trial-level data, and analysis files for all experiments are available via the Open Science Framework: https://doi.org/10.17605/OSF.IO/Q4PGH. The study protocol was not preregistered.

**Supplementary Information:**

The online version contains supplementary material available at https://doi.org/10.3758/s13414-026-03342-4.

## Introduction

Studying how eye movement control develops in children is important to understanding their abilities in educational contexts as well as with respect to atypical development (e.g., Hodgson et al., 2025; Sumner et al., 2018). An important aspect of performing many complex tasks is the ability to exert cognitive control and inhibit stimulus driven saccadic eye movements (Findlay & Gilchrist, 2003; Hodgson et al., 2019). A test that has been widely used to study this aspect of eye movement control in adults and nonhuman primates is the oculomotor stop signal or countermanding saccade task (Hanes & Carpenter, 1999; Hanes & Schall, 1995), but few studies to date have examined performance of this task in children. The current study presents a detailed investigation of the countermanding task in a large sample of typically developing primary-school-age children with the aim of assessing how performance in the task develops with age.

The countermanding task was developed for use in nonhuman primates as an oculomotor version of manual response key stop signal tests (Hanes & Carpenter, 1999; Hanes & Schall, 1995; Lappin & Eriksen, 1966; Logan & Cowan, 1984). Participants make saccades towards targets appearing to the left or right of central fixation. On some trials a stop signal can appear shortly after target onset (usually a change in colour or reappearance of the central fixation stimulus), which instructs participants to inhibit responding and “countermand” the saccade towards the peripheral target. The stop signal delay (SSD) between the onset of the target and the stop signal is typically varied across trials. The task differs from the other widely used test of inhibitory oculomotor control, the anti-saccade task (Hallet & Adams, 1980), due to its demand to implement reactive inhibition following the target onset on a subset of trials. Participant performance is characterised by two types of errors: omission errors, in which participants fail to make a saccade within the “Go” trial response window, and commission errors*,* where a saccade is made to the target on a “Stop” trial.

By examining the proportion of errors and reaction times on different trial types, insights can be drawn into the neural and cognitive processes mediating response inhibition. Commission errors have been reported to increase with SSD, suggesting that there is a limited time window within which a programmed saccade can be successfully cancelled. The most widely applied model of the task assumes a horse-race-like competition between two independent internal processes (Hanes & Carpenter, 1999; Logan, 1983; Verbruggen & Logan, 2009). A go process initiated by the target rises towards a threshold level at which point a saccade is triggered. Simultaneously, a stop process can be initiated by the stop signal onset. If the stop process reaches threshold first, the go process is cancelled and no response is executed, whereas if the go process wins, a commission error occurs. Through examination of the change in the proportion of errors occurring at different SSDs, an estimate of stop signal reaction time (SSRT) can be calculated, which equates to the average time required to successfully cancel a programmed saccade. Potential neural substrates of the stop and go processes have also been identified in the frontal cerebral cortex of Macaque monkeys (Hanes et al., 1998) where activity of cells tuned to saccade preparation and active fixation are found.

The horse-race model and stop signal tasks in general have been highly influential, including in the study of neurological, behavioural and developmental disorders (Aron et al., 2007; Lijffijt, et al., 2005), although alternative perspectives have suggested more complex interacting influences on stop signal task performance other than inhibitory control (e.g., Bissett et al., 2021; Liddle et al., 2009; Özyurt et al., 2003). The performance of countermanding saccades in children is of interest in the light of these debates, as well as for understanding the development of eye movement control in everyday and educational contexts.

The ability to exert inhibitory control over sensorimotor reflexes is assumed to develop through childhood and into adolescence as the frontal and parietal cerebral cortices mature (Velanova et al., 2008; Williams et al., 1999). At birth, saccades and fixation are reported to be driven exclusively towards salient objects in the visual field, but this is followed by a period when babies are reported to be overly engaged on visual objects and have great difficulty disengaging fixation (Johnson et al., 1991; Stechler & Latz, 1966). Whilst stimulus directed saccades can often be difficult to elicit and record in babies (Harris et al., 1993), the fast phase of optokinetic nystagmus has been shown to have normal spatiotemporal dynamic characteristics, indicating that the neural circuitry underlying saccade execution is mature soon after birth (Garbutt et al., 2006). Errors in the anti-saccade task have been consistently shown to reduce with increasing age in children (Bucci & Seassau, 2012; Hodgson et al., 2025; Sumner et al., 2018) and progressive development of stopping ability has been demonstrated in manual stop signal tasks in children aged 6 years and older (Williams et al., 1999). A similar developmental trajectory would therefore be expected for the countermanding saccade task. Only two published studies to our knowledge have applied the task in primary-school-aged children, and these have used either a very narrow age range of children over 10 years old or included children with adults as part of a mixed-age control group to compare with an atypically developing group (Hanisch et al., 2006; Kelly et al., 2021). As such, a comprehensive analysis of developmental effects in the task has not been reported. With respect to the horse-race model, it is predicted that the stop process should be slower to reach threshold, highly variable, or completely absent in younger children. This should be apparent as high rates of commission errors in younger children and a negative association between stop trial errors and increasing age.

In Experiment 1, children aged 3–11 years completed a child-friendly version of the countermanding task, in which a cartoon bee jumped from the centre to the left or right of the screen and a green or a red flower presented at fixation served as the go/stop signal. We anticipated that younger children would be poorer than older children at inhibiting saccades towards the target bee on stop trials, such that older children would make fewer commission errors in the task.

## Experiment 1

### Methods

#### Participants

The study was carried out at an annual Summer Scientist Week (SSW) event, a public participation and involvement in research event at the University of Lincoln for primary-school-aged school children and their parents. At the event, research studies are presented as fun games, and children earn tokens for completing the “science games”, which they can then spend on other activities such as face painting and Mario Kart racing.

Eighty-seven out of the 200 children attending the event played the Busy Bee game, with 53 out of the 87 children completing the task with sufficiently good eye-tracking recordings for inclusion in the analysis, such that saccades could be clearly identified on more than 50% of trials without blink interference or loss of signal. The children who completed the task ranged in age from 3.38 to 10.89, had mean age of 7.08±1.93 years, and a median age of 6.78 years. One previous study has reported a significant effect of age on Stop trial errors in the countermanding saccade task across a mixed aged sample including both children and adults (Kelly et al., 2021). Based upon the effect size reported in this previous work (*f*^2^ = 0.25), our study had an estimated statistical power of 1 − β = 0.98 for detecting an effect of age on Stop trial errors at α = 0.05 (G*Power, Version 3.1.9.7; Faul et al., 2007). Statistical analysis was carried out using SPSS (Version 29.0) and JASP (Version 0.19.3.0). Classical and Bayesian analyses of variance (ANOVAs) were conducted for effects of interest. For each main effect and interaction effect inclusion Bayes factors (*BF*_*incl*_) are reported, indicating how much more likely models which include the effect are relative to models that do not include the effect. Bayes factors >1 indicate evidence against the null hypothesis with values between 0.3 and 3 typically considered to be inconclusive evidence for or against. For ANOVAs using age as a factor, children were divided into three groups with approximately equal numbers of children within each group: 3 to 6 years (*N* = 19); 6 to 8 years: (*N* = 18); 8+ years (*N* = 16). Exact monthly age was also used to assess associations between dependent measures via Pearson correlations.

#### Task and procedure

The Busy Bee game was programmed using Experiment Builder software (Version 2.3.38) together with an EyeLink 1000 eye-tracking system (SR Research Ltd) configured in remote head free mode. In this configuration, a target sticker attached to the participant’s head is used to compensate for head movements. Stimuli were displayed on a ViewSonic LCD monitor refreshing at 120 Hz. Children were asked to “keep still like a statue” while the eye tracker was being set up and during the game itself to reduce head movements. A three-location triangular calibration grid was used as it allowed for rapid and accurate horizontal and vertical calibration.

The task comprised a short block of six practice trials, followed by a main block of 40 trials, 16 of which were Stop trials. A high proportion of Stop trials can lead to more strategic slowing of responding to avoid commission errors. In choosing the trial ratio of 40% of trials we aimed to balance the requirement for Stop trials to comprise a minority of trials (Verbruggen et al., 2019) whilst ensuring sufficient Stop trials to obtain estimates of error rate and saccade reaction time (SRT) within a short test block. Similar Stop trial ratios have been used in several published studies of the countermanding task (e.g., Kelly et al., 2021). Trials were presented in a different pseudo-randomised order across participants, as determined by Experiment Builder’s randomisation protocol. No run length control was used for randomisation of target location (left/right) or trial type (Go/Stop), but analysis showed that 58.7 of Stop trials across the entire dataset were preceded by a Go trial, with only 1.7% of Stop trials preceded by a run of two or more consecutive Stop trials.

**Fig. 1:**
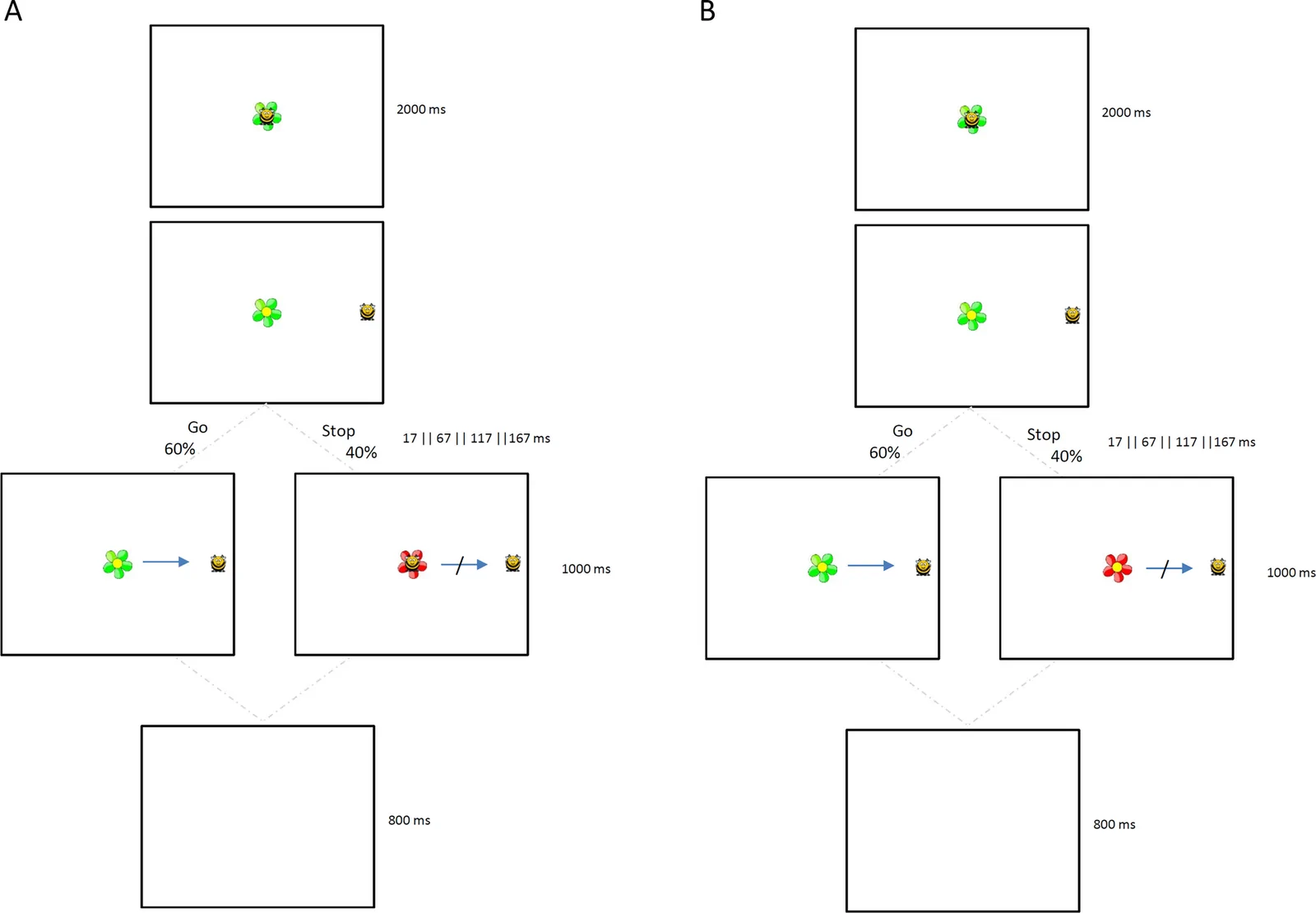
Schematic illustration of the trial sequence used in (**A**) Experiment 1 and (**B**) Experiment 3. Experiments 1 and 2 differed only with respect to the instructions given to participants regarding the meaning of the red flower (stop signal). Experiment 3 differed from Experiment 1 with respect to the removal of the bee overlapping the red flower on Stop trials

At the start of each trial a cartoon bee (subtending approximately 1° of visual angle) was displayed overlaid on top of a green cartoon flower (2°) at the centre of the screen for 2,000 ms. The bee was then extinguished and redisplayed at either 15° to the left or the right of the target. This eccentricity value was chosen to ensure reliable eye movement response detection and triggering, such that leftward and rightward target saccades were clearly identifiable and robust to calibration drift. Although larger than most other studies of the countermanding task, target eccentricities of 15°° have been used in previous studies with humans and Macaques with no apparent effect on performance (e.g., Colonius et al., 2001; Hanes & Schall, 1995). On Go trials, the green flower continued to be displayed at fixation for a further 1,000 ms (the response window). On Stop trials, the peripheral target remained on the screen until the end of the response window, but following a variable stop signal delay (SSD) the central flower changed colour from green to red, and a cartoon bee reappeared on top of the flower (Fig. 1A). Experiment Builder software ensures display updates are precisely synchronised to screen refresh rates and stimulus timing was verified by time stamped event markers within the eye movement data file. Actual values of SSD (as opposed to the values specified in the Experiment Builder script) were confirmed to the nearest ms from the time-stamped event markers within the eye movement data file as 17, 67, 117, and 167 ms, with four trials of each SSD presented within the test block. Correct responses were followed by a system “beep” sound effect (Windows media “notify.wav” sound) and errors (whether commission or omission) were followed by a higher pitch “alert” sound effect (Windows media “speech_misrecognition.wav” sound).

Instructions were displayed on the screen prior to the start of the experiment as well as following the practice block and were also read out loud to children as follows:

*“Follow busy bee with your eyes as she jumps around the screen. Sometimes the flower is green and sometimes its* [sic] *red. If the flower stays green follow busy bee, but if the flower turns red look straight ahead instead”.*

#### Eye movement analysis

Eye movements were analysed using SR Research Dataviewer software (Version 4.3.1). Saccades were defined as periods where the eye velocity exceeded 30°/s with acceleration exceeding 80,000°/s^2^. The first detected saccade in the direction of the target which exceeded 1.5° amplitude following the target onset was classified as a trial response, although 98% of actual trial responses recorded had amplitudes of 5° or greater. In cases where only saccades smaller than 1.5° were detected during the response window, trials were classified as omission errors or successful stop trials. Trials where a response was detected between 200 ms prior to and 70 ms after the target onset were considered anticipatory errors and were excluded from the analysis. Anticipatory errors comprised only 0.6% of all trials in Experiment 1 and were similarly low in Experiment 2 (0.8%) and Experiment 3 (2%) such that they were not subject to further statistical analysis.

### Results

#### Response errors

**Fig. 2:**
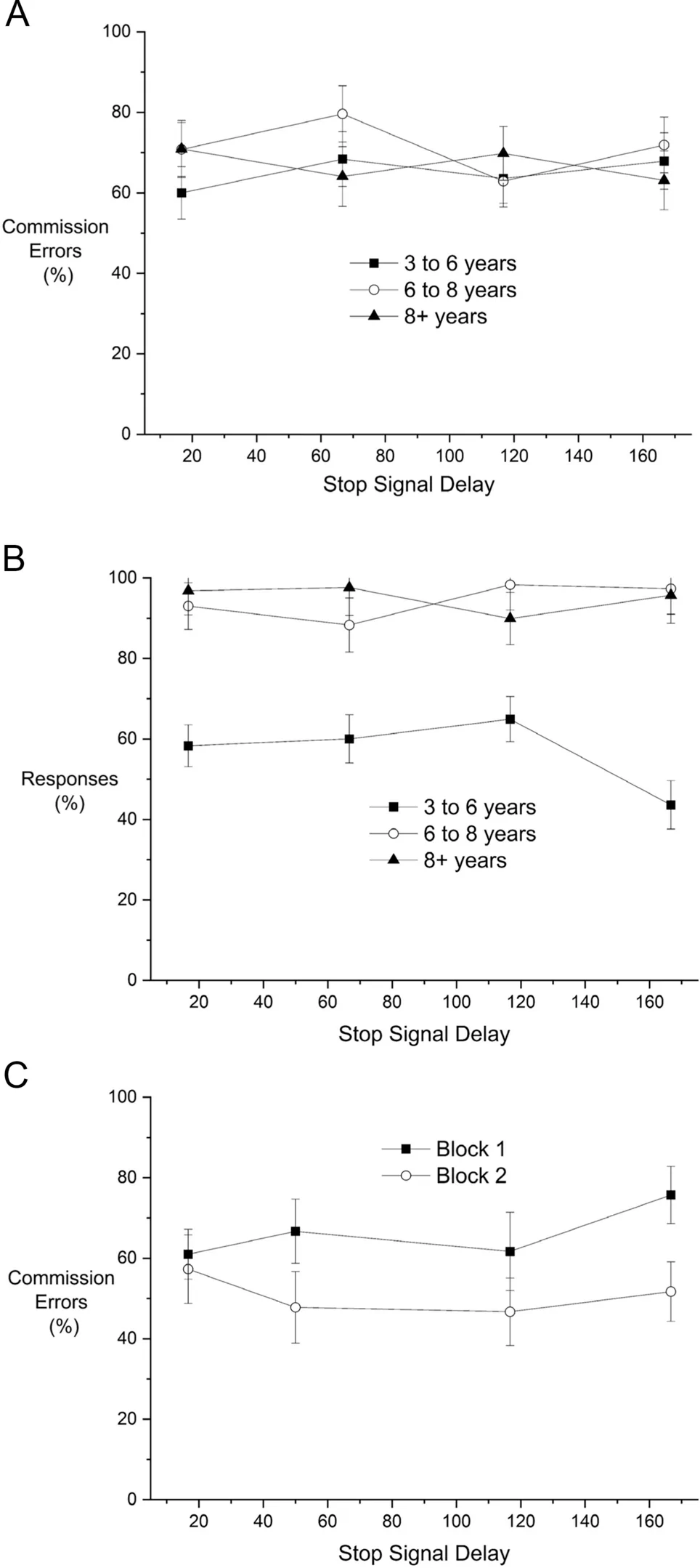
Percent commission errors (Experiments 1 and 3) and response rate (Experiment 2) on Stop/Red flower trials, plotted against SSD for (**A**) Experiment 1, (**B**) Experiment 2, and (**C**) Experiment 3 (error bars indicate standard error)

**Fig. 3:**
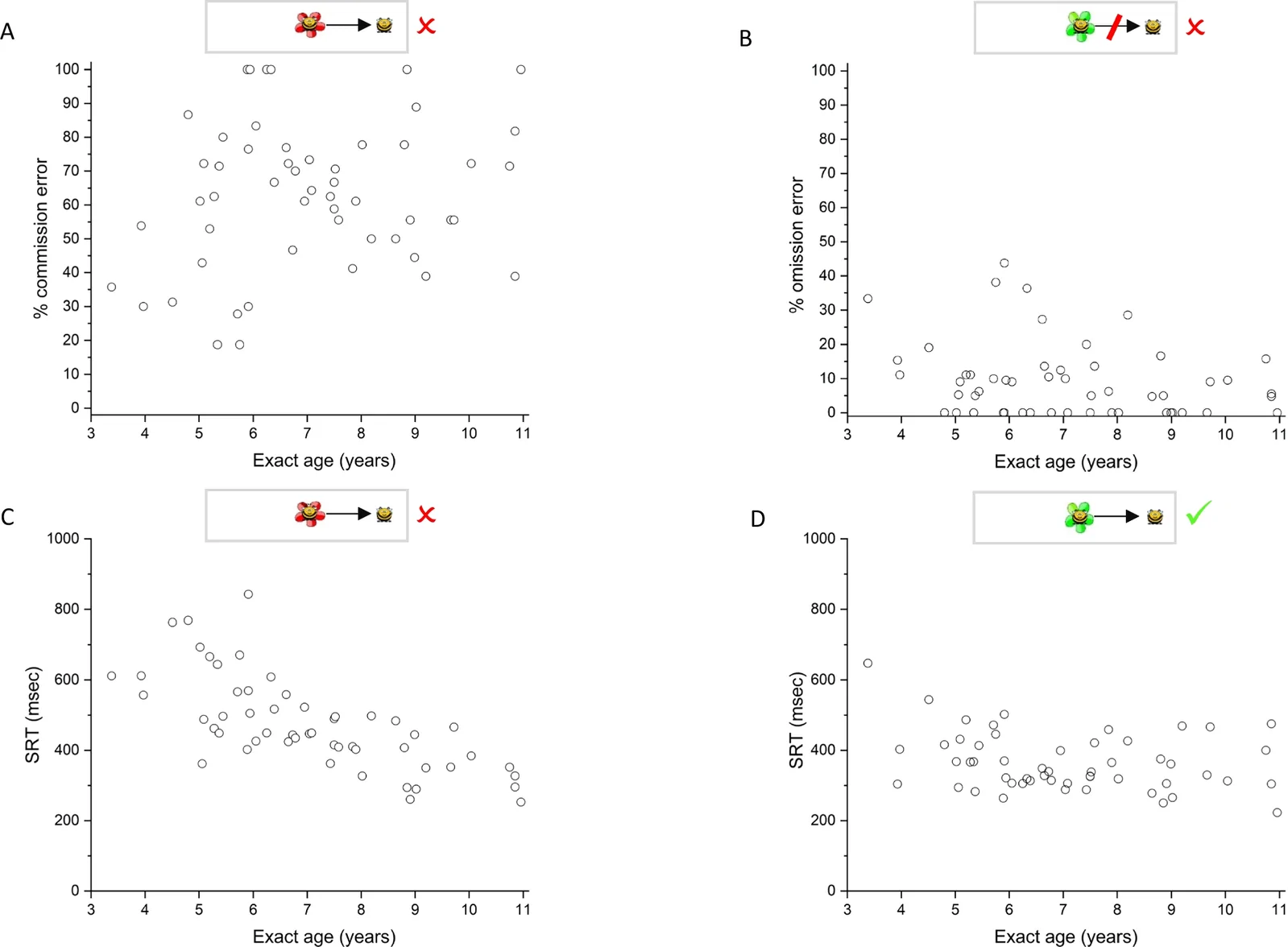
Scatter plots of performance against age for Experiment 1. **A** Commission errors on Stop trials. **B** Omission errors on Go trials. **C** SRTs of errors on Stop trials. **D** SRTs of correct responses on Go trials

*Stop trials.* Errors of commission, where children made a saccade to the target, occurred on 66% of Stop trials averaged across all participants. There was no significant effect of Age group on commission errors, *F*(2,50) = 0.439, *p* = 0.647, η_p_^2^ = 0.017, *BF*_*incl*_ = 0.188 (Fig. 2A; Fig. 3A). Neither was there a significant effect of SSD, *F*(3,150) = 0.509, *p* = 0.677, η_p_^2^ = 0.01, *BF*_*incl*_ = 0.048, or interaction between Age and SSD, *F*(6,150) = 1.097, *p* = 0.367, η_p_^2^ =0.042, *BF*_*incl*_ = 0.128 (Fig. 2A).

*Go trials.* Errors of omission, where children failed to make a saccade towards the target on Go trials within the response window averaged 8.9% across all participants. There was no significant effect of Age on errors of omission, *F*(2,50) = 1.224, *p* = 0.303, η_p_^2^ = 0.047, *BF*_*incl*_ = 0.354 (Fig. 3B).

#### Saccade amplitude gain

The amplitude gain of saccades was defined as the ratio between the amplitude of a saccade response and the eccentricity of the target stimulus, with 1.0 corresponding to perfectly accurate saccade and values <1.0 corresponding to saccades undershooting the target. Analysis showed no significant main effect of Trial Type, *F*(1,50) = 2.23, *p* = 0.141, η_p_^2^ = 0.043, *BF*_*incl*_ = 0.623, Categorical Age, *F*(2,50) = 2.80, *p* = 0.070, η_p_^2^ = 0.101, *BF*_*incl*_* =* 1.02, or interaction between Age and Trial type on saccade gain, *F*(2,50) = 0.151, *p* = 0.860 η_p_^2^ = 0.006, *BF*_*incl*_ = 0.157. A significant positive correlation was observed between exact age and amplitude gain on Go trials, ρ = 0.336, *p* = 0.014, but not for Stop trial errors, ρ = 0.194, *p* = 0.164.

#### Saccade reaction times

Saccade Reaction times (SRTs) for Stop trial errors were significantly longer than correct Go trial responses, *F*(1,50) = 70.01, *p* < 0.001, η_p_^2^ = 0.583, *BF*_*incl*_ = 2.346×10^7^ (mean Go: 364 ms versus Stop: 469 ms). There was a significant effect of Age with younger children showing slower SRTs, *F*(2,50) = 16.56, *p* < 0.001, η_p_^2^ = 0.398, *BF*_*incl*_ = 5229.702, and a significant interaction between Trial Type (Go/Stop) and the effect of Age, *F*(2,50) = 14.72, *p* < 0.001, η_p_^2^ = 0.371, *BF*_*incl*_ = 1860.588. This interaction was reflected in a stronger negative correlation between SRTs and exact age for Stop compared with Go trials (Pearson correlations Stop: ρ = 0.709, *p* < 0.001 versus Go: ρ = 0.280, *p* = 0.042; Fig. 3C–D).

SRTs on Go trials also correlated positively with rates of omission errors across participants, ρ = 0.459, *p* < 0.001, and negatively with stop trial errors, ρ = −0.616, *p* < 0.001. SRTs for Stop trial error responses correlated positively with rates of omission errors on Go trials, ρ = 0.478, *p* < 0.001 and negatively with rates of commission errors on Stop trials, ρ = −0.363, *p* = 0.007.

#### Reaction time variability and distributions

**Fig. 4:**
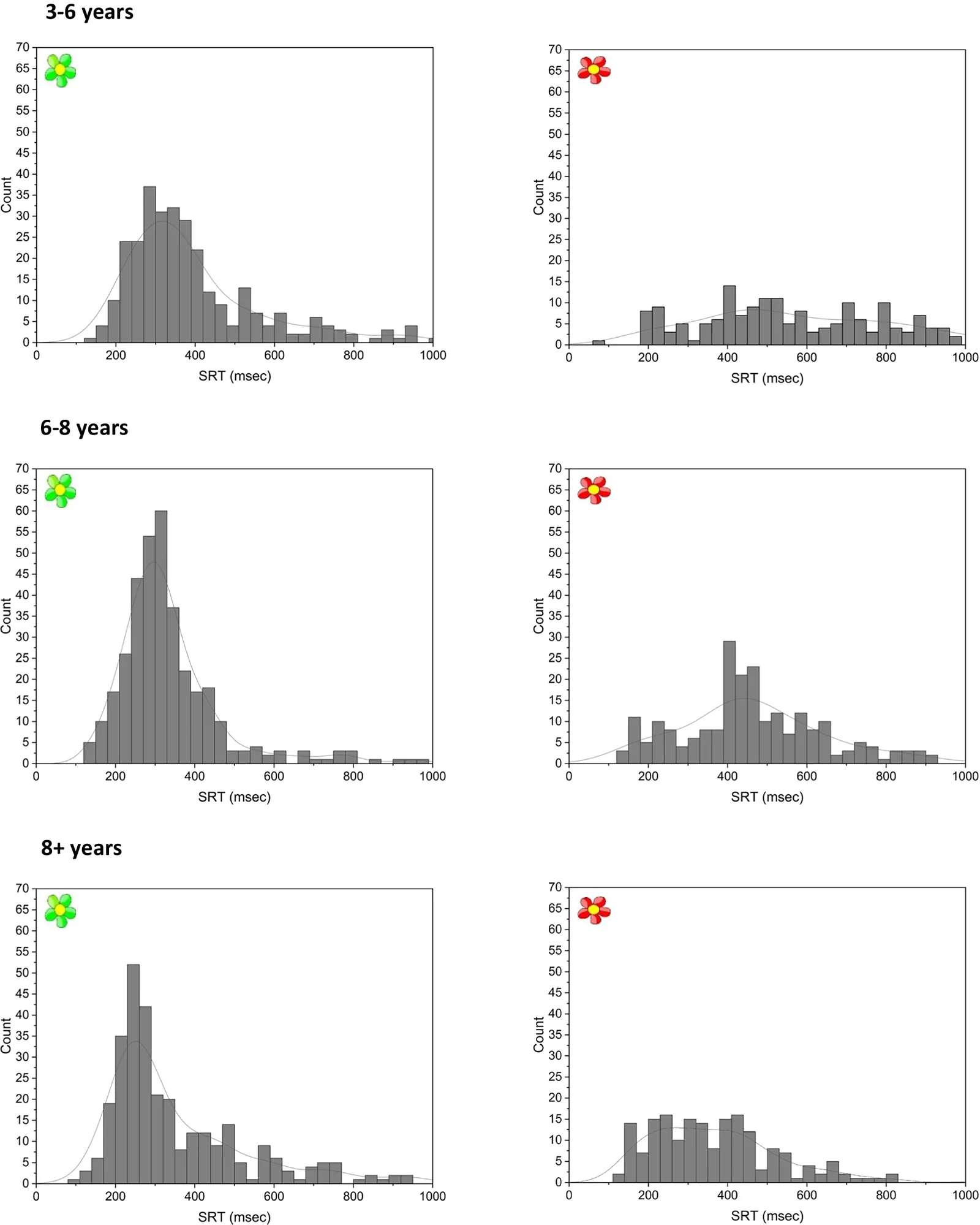
Compound target onset locked SRT distributions for correct Go trials (left panel) and Stop trial errors (right panel) for the different age groups in Experiment 1 (with spline function fit lines)

A two-way ANOVA with Trial Type and categorical Age as factors examined standard deviations of SRTs across participants. This showed a significant main effect of Trial Type, *F*(1,50) = 13.36, *p* < 0.001, η_p_^2^ = 0.21, *BF*_*incl*_ = 58.403, and Age on SRT variability, *F*(2,50) = 5.65, *p* = 0.006, η_p_^2^ = 0.184, *BF*_*incl*_ = 6.011, with the mean variability being increased for Stop relative to Go trials and reducing with Age. There was a two-way interaction between Trial Type and Age, *F*(2,50) = 7.65, *p* = 0.001, η_p_^2^ *=* 0.234, *BF*_*incl*_ = 40.053, suggesting that the increased variability of Stop trials was larger in younger children. Examination of compound reaction time distributions (formed by aggregating SRTs across all participants within an age group), confirmed that whilst younger children made some Stop trial errors with very short SRTs (<100 ms), they also had a more extended “tail” of very long SRTs compared with older children (Fig. 4).

### Discussion

The findings of Experiment 1 were surprising relative to our prior expectations and hypotheses. We had anticipated that the youngest children would find it very difficult or be totally unable to countermand stimulus driven saccades, but this was not the case. Even some of the youngest children (3–6 years) appeared to be able to countermand saccades on a proportion of Stop trials (averaging 44% correct responses). Although some children made 100% Stop trial errors, these were found to be distributed across the entire age range and no significant effect of age on commission errors was found (Fig. 2A; Fig. 3A). Another unexpected result was that mean SRTs for Stop trial errors were significantly longer than Go trials. This finding is problematic for horse-race models, as errors of commission should represent the lowest end of the typical SRT distribution and so should always be quicker on average than Go trial responses (Bissett et al., 2021). Stop signal errors also did not increase with SSDs. This is perhaps due to the fixed range of SSD used and the very low trial numbers necessitated by working with young children, although we had expected to see some variation in commision errors with SSD when averaging across participants. The finding of no effect of SSD could be interpreted as implying either a highly variable stop process in children, or one that it is best considered as a binary-stochastic process, related to failures to attend to the stop signal (also termed inattention or “trigger failures”; Bissett et al., 2021). Although it is possible to estimate individual participants’ SSRTs in the absence of an effect of SSD (using the “integration method”; Logan & Cowan, 1984), we did not consider this analysis justified given the extent to which the data deviated from the predictions of the race model (though see Experiment 3).

One aspect of children’s performance for which there was a clear effect of age was the increase in SRTs for Stop relative to Go trials, which was much larger in younger children (Fig. 3C–D). Inspection of response distributions revealed very long SRT errors on Stop trials in younger children with responses occuring right up until the end of the 1,000-ms response window (Fig. 4). One explanation for this might be that children found it difficult to sustain inhibition throughout the trial period, such that whilst a response might be delayed, it was eventually initiated once sustained endogenous inhibition fails. This would predict an accumulating frequency distribution of SRTs with more errors occurring towards the end of the response window. An accumulating pattern was not observed and the SRT distribution for stop trial errors was very flat in the youngest group (Fig. 4), although failures to sustain attention cannot be completely excluded as this pattern may be masked when aggregating distributions across participants. Another explanation for long SRT Stop trial errors is that the stop signal had a transient exogenous inhibitory effect on saccade generation, which delayed rather than completely suppressing saccade execution. Recent research and modelling of oculomotor task performance in adults has shown that non-target stimulus onsets generate a transient inhibitory effect on saccade generation, leading to “dips” in observed SRT distributions (Bompas et al., 2020). In the case of the countermanding task, the appearance of the stop signal at fixation might directly inhibit saccade generation. This exogenous effect is transient and involuntary in nature such that it occurs even when no instruction is given to countermand saccades.

In order to isolate this potential exogenous inhibitory effect of the stop signal, the same task was presented to a new group of children who were given different instructions. This time the instruction was to follow the bee regardless of whether the flower was green or red. All other stimulus presentation variables were kept constant, so that any differences in performance between Experiments 1 and 2 must be attributable to the influence of endogenous inhibitory control processes, whereas similarities would reflect exogenous effects of the stop signal.

## Experiment 2

### Methods

#### Participants

Fifty-seven children at another SSW event completed Experiment 2, of which 48 completed at least 24 trials and had eye data of sufficient quality to analyse. None of the participants had completed the original version of the task. Age range was 3.0–10.66 years with a mean age of 6.75±2.18 years and a median age of 6.76 years. To faciliate comparison with Experiment 1 the same age bands were used in the categorical (ANOVA) analyses, with participant numbers in each group as follows: 3 to 6 years (*N* = 19); 6 to 8 years: (*N* = 15); 8+ years (*N* = 14).

#### Task and procedure

The task and procedure were identical to Experiment 1 except for the following: Rather than being instructed to look straight ahead when the flower turned red, this time children were instructed as follows:

*“Sometimes the flower is green and sometimes it’s red, but you just follow busy bee instead”.*

In Experiment 2 we refer to Go and Stop trials as “Green flower” and “Red flower” trials, as the colour of the flower had no instructional value. For consistency we still refer to the delay between the target onset and the “stop” signal as SSD in the Results and Discussion.

### Results

#### Response omission errors

**Fig. 5:**
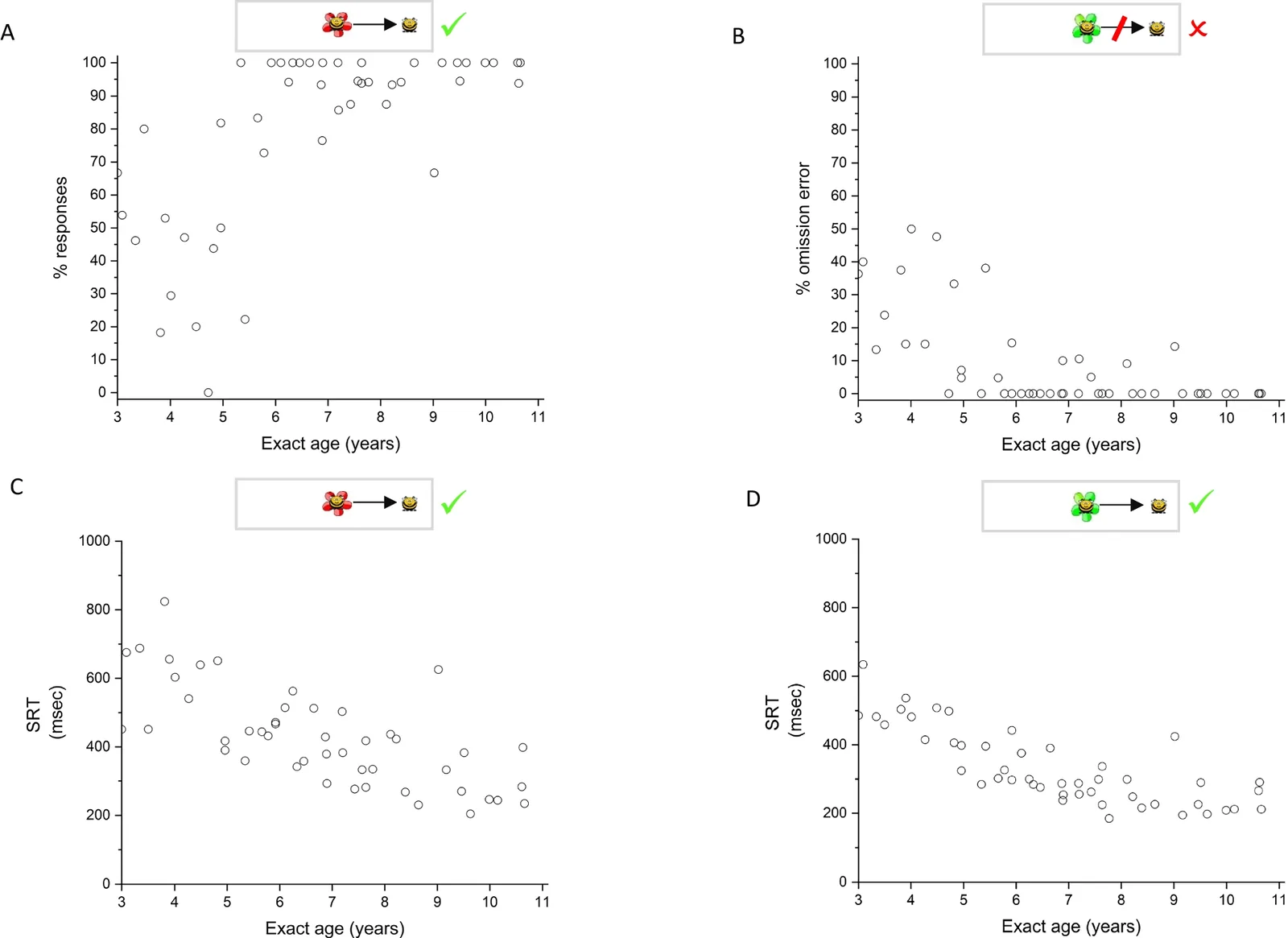
Scatter plots of performance against age for Experiment 2. **A** Responses on Red flower trials. **B** Omission errors on Green flower trials. **C** SRTs of responses on Red flower trials. **D** SRTs of responses on Green flower trials

Although there was no requirement to countermand saccades in Experiment 2, response omissions were frequently observed, with 9.4% of Green flower trials and 21.1% of Red flower trials being omission errors. A significant main effect of Age was found on response omissions, *F*(2,45) = 20.50, *p* < 0.001, η_p_^2^ = 0.477, *BF*_*incl*_ = 0.130, with children aged 3–6 years responding on only 57% of Red flower trials, compared with 95% of trials for children aged 6 years and over. There was also a significant interaction between Age and SSD, *F*(6,135) = 2.89, *p* = 0.011, η_p_^2^ = 0.114, *BF*_*incl*_ = 4.717, with the youngest children making *more* omission errors at the longest SSDs, Fig. 2B; Fig. 5A. Errors of omission on Green flower trials also showed a significant effect of Age, *F*(2,45) = 14.76,*p* < 0.001, η_p_^2^ = 0.396, *BF*_*incl*_ = 1797.565, with the youngest age group making 20% omission errors, compared to 2% for 8–12 year old children (Fig. 5B).

#### Saccade amplitude gain

Saccade amplitude gain was significantly lower on Red flower trials compared to Green flower trials, *F*(1,44) = 4.81, *p* = 0.034, η_p_^2^ = 0.099, *BF*_*incl*_ = 1.038 (means Red = 0.925; Green = 0.948), but there was no main, *F*(2,44) = 0.720, *p* = 0.492, η_p_^2^ = 0.032, *BF*_*incl*_ = 0.336, or interaction effects, *F*(2,44) = 1.778, *p* = 0.181, η_p_^2^ = 0.075, *BF*_*incl*_ = 0.568, of categorical Age. There was also a main effect of SSD, with saccade gains decreasing significantly with SSD, *F*(3,108) = 3.874, *p* = 0.011, η_p_^2^ = 0.097, *BF*_*incl*_ = 4.23 (means 17 ms = 0.957; 60 ms = 0.946; 117 ms = 0.911; 167 ms = 0.895).

#### Saccade reaction times

Mean SRTs were longer on Red compared with Green flower trials, *F*(1,44) = 75.93, *p* < 0.001, η_p_^2^ = 0.63, *BF*_*incl*_ = 2.575×10^8^ (mean Green: 320 ms versus Red: 418 ms). There was a significant effect of Age on SRTs, *F*(2,44) = 20.469, *p* < 0.001, η_p_^2^ = 0.48, *BF*_*incl*_ = 28525.912, but no interaction between Trial Type and Age, *F*(2,44) = 0.887, *p* = 0.419, η_p_^2^ = 0.039, *BF*_*incl*_ = 0.398. Significant negative correlations were found between exact age and SRTs for both trial types (Red flower: Pearson −0.71, *p* < 0.001; Green Flower: Pearson = −0.79, *p* < 0.001; Fig. 5C–D).

#### Saccade reaction time variability

As with Experiment 1, younger children showed increased variability in SRTs, *F*(2,44) = 10.78, *p* < 0.001, η_p_^2^ = 0.329, *BF*_*incl*_ = 124.4, and there was also a main effect of Trial Type with Red flower trials showing greater variability than Green flower trials, *F*(2,44) = 26.06, *p* < 0.001, η_p_^2^ = 0.372, *BF*_*incl*_ = 15995.171, but no interaction between Trial Type and Age, *F*(2,44) = 0.289, *p* = 0.751, η_p_^2^ = 0.013, *BF*_*incl*_ = 0.202.

#### Analysis of “dips” in SRT distribution

Further analysis was carried out to determine if transient inhibitory effects of the “stop signal” were apparent in SRT distributions using an approach based upon the one-sample chi-squared (χ^2^) or “goodness-of-fit” procedure for analysing frequency distributions (Waddington & Harris, 2013). SRTs were first grouped into time bins of width determined via the Friedman–Diaconis optimisation rule (Freedman & Diaconis, 1981). Expected frequency values within each time bin on Red flower trials were then predicted based upon the total observed distribution of SRTs across both trial types as follows:

$$Ex{p}_{Stop\left(current\, time bin\right)}=\frac{Ob{s}_{Stop\left(all \,time \,bins\right)}\times Ob{s}_{Total\left(current \,time \,bin\right)}}{Ob{s}_{Total\left(all\, time \,bins\right)}} ,$$

where *Exp_Stop* is the expected number of responses within a given time bin on Red Flower trials; *Obs_Stop* is the actual observed number of responses on all Red Flower trials, and *Obs_Total* is the total number of responses recorded across both trial types. To derive an estimate of statistical significance of the deviation from expected value within each time bin, the standard *χ*^*2*^ equation was rearranged, substituting the critical value of χ^2^ (${{\chi }^{2}}_{crit}$) to calculate the associated difference between expected (*Exp*) and observed (*Obs*) frequencies:

#### ${\left(Obs-Exp\right)}_{crit}=\pm \sqrt{Exp\times {{\chi }^{2}}_{crit}}$*.*

**Fig. 6:**
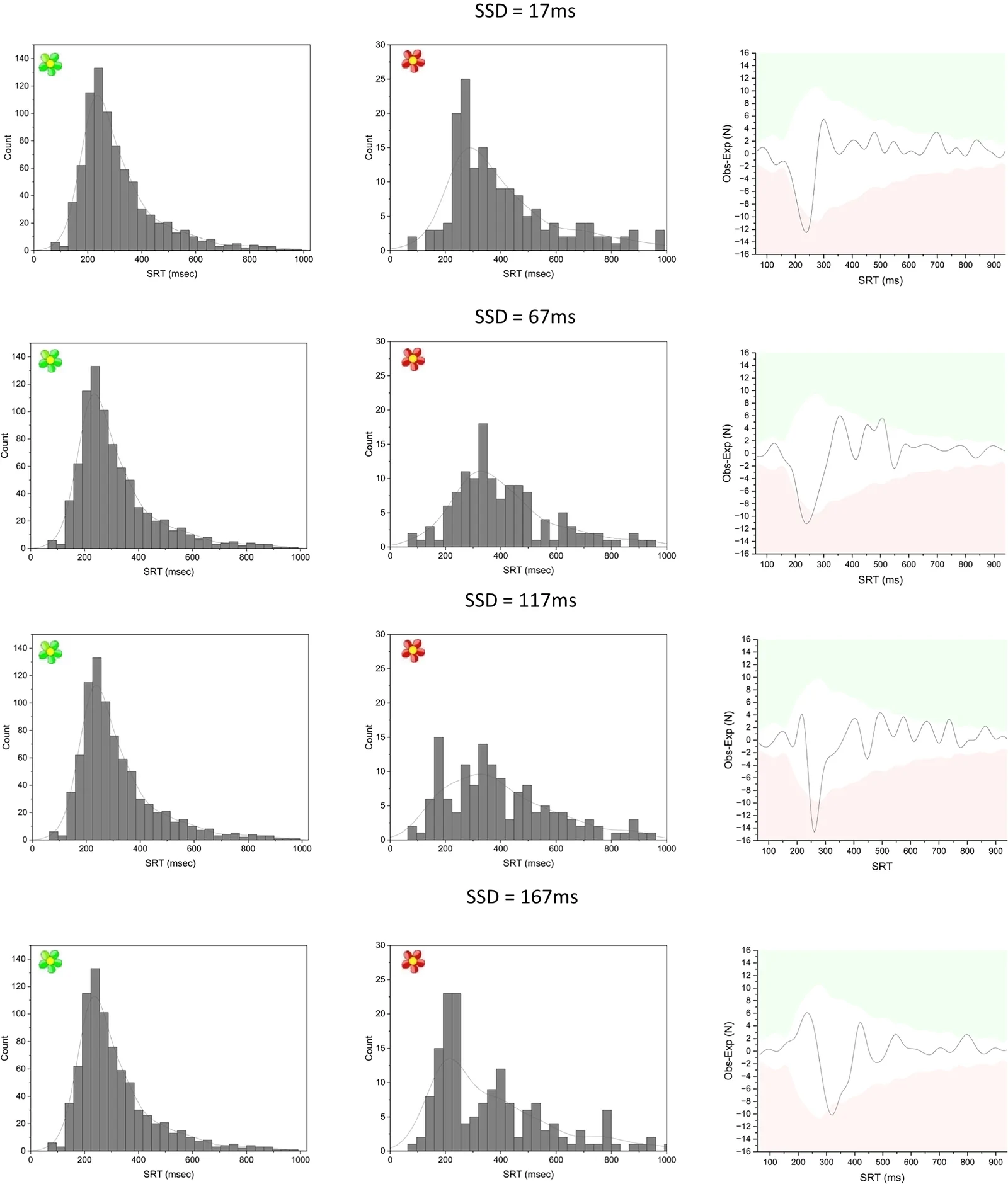
Red flower trial (middle panels) target onset locked SRT distributions and observed versus expected values (right panels) with fitted spline functions in Experiment 2 for the 4 SSD values (all ages combined). Shaded regions show estimated critical values of observed versus expected value difference across SRTs (based on Χ^2^ = 5.025, *df* = 1, *p* < 0.025). The distribution of SRTs for all Green flower trials is shown in each case (left panels) to aid direct visual comparison of distributions

**Fig. 7:**
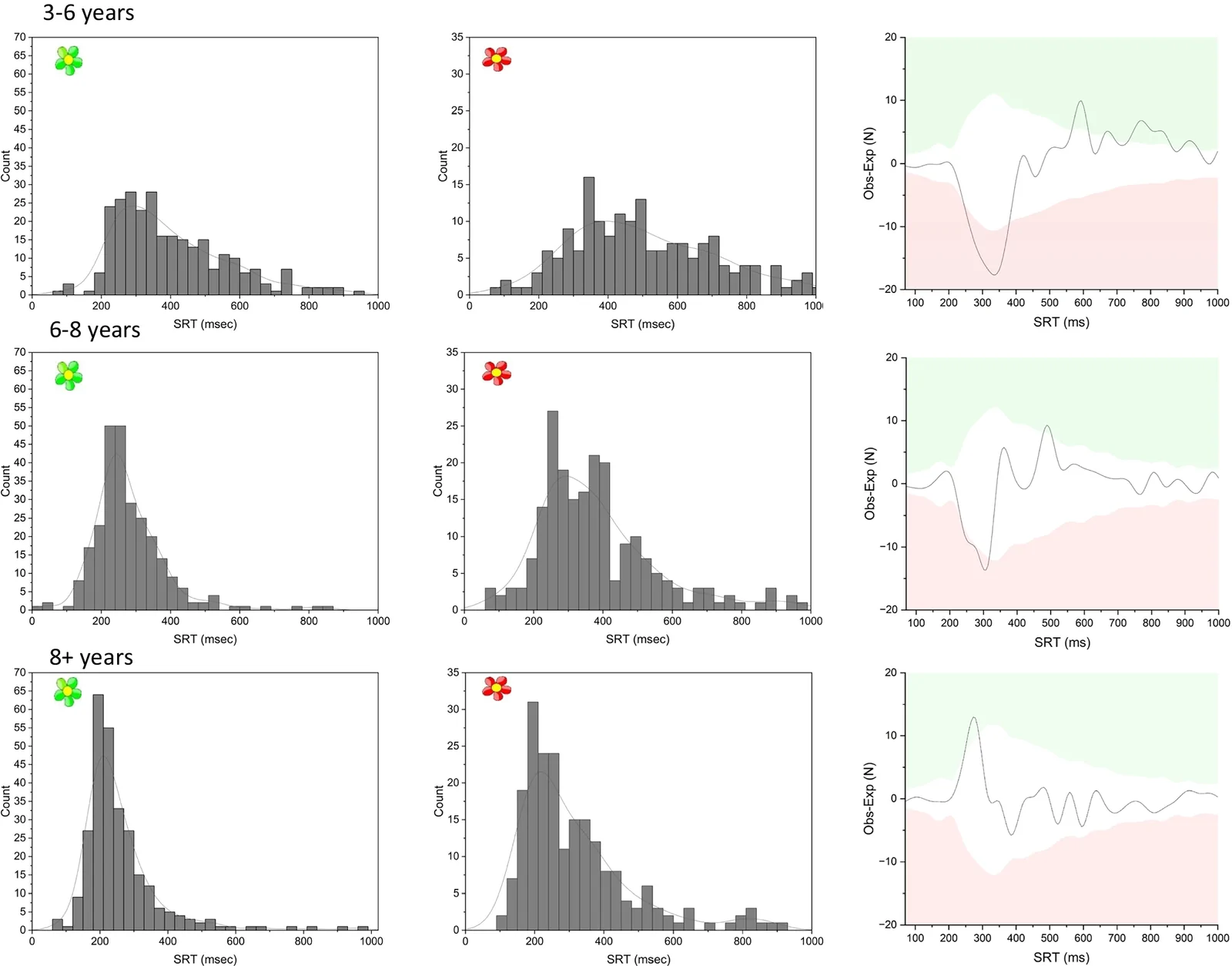
Green and Red flower trial target onset locked SRT distributions (left and middle panels) and observed versus expected values (right panels) in Experiment 2, for different age groups. Shaded regions show estimated critical values of observed versus expected value difference

This analysis is summarised in Figures 6 and 7. For each SSD condition, a dip was observed in the target onset locked SRT distributions 100–150 ms after the red flower onset such that it occurred later at longer SSDs (Fig. 6). Comparing age groups and collapsing across SSD conditions, similar dips were apparent for younger children, but not for the oldest group, who instead showed an increase in expected frequencies (i.e., a “blip”) in the distribution (Fig. 7).

### Discussion

Analysis of children’s SRTs and omission errors in Experiment 2 confirmed that the stop signal exerts an exogenous inhibitory effect on saccade generation. The youngest age group made a similar number of response omissions on Red flower trials to that seen in Experiment 1, even though no instructions were given to countermand saccades. SRTs were again found to be significantly longer on Red flower relative to Green flower trials and “dips” were observed in SRT distributions, peaking 100-150ms after the onset of the stop signal (Figs. 6 and 7). This effect is consistent with computational models which propose a transient inhibitory influence of non-target stimulus onsets in oculomotor paradigms (Bompas et al., 2020; Trappenberg et al., 2001). The dip in SRT distributions was found to be clearest in younger children (<8 years), whereas for the older children a “blip” was seen relative to the expected SRT frequency distribution shortly after the stop signal (Fig. 7). It was suggested above (Discussion, Experiment 1), that very long SRT stop trial errors in younger children might reflect difficulty sustaining attention and fixation throughout the response window, but the finding of similarly long response times for 3 to 6 years olds on Red flower trials in Experiment 2 (Fig. 7) indicates that this is unlikely to be the case. Instead, it is more likely that the exogenous inhibitory effect of the stop signal led to long SRT stop trial errors in Experiment 1. Also consistent with an exogenous effect of the stop signal on saccade programming, saccade amplitude (i.e., size) was reduced on Red flower trials in Experiment 2, and this effect decreased with SSD, suggesting stop signals can also directly effect the spatial parameters of saccades even when they have no instructional value.

Although dips in SRT distributions relative to expected frequency have been found in previous studies in adults (Bompas et al., 2020), previous work has not reported “blips” in the frequency of responding. It is possible that the chi-squared procedure used here may be better at detecting positive deviations from expected frequency than visual inspection of distributions alone (particularly in the early and late part of the distribution where response frequencies are low). Where a dip reflects a delay in responding, increases in frequency would be expected later in the distribution as seen for children under 8 years here (Fig. 7, upper panels), but the blip in the early part of the distribution in children over 8 years is more puzzling (Fig. 7, bottom panel). A very speculative explanation is that the red flower had a facilitatory effect on responding in older children, either due to an attentional alerting effect or as a strategy to accelerate responding to overcome the disruptive effect of the red flower on saccade programming.

In our analysis of SRT distributions by age, trials with different SSDs were pooled together. This was done as splitting the distributions by both Age and SSD would lead to insufficient trials in each condition to indentify changes in expected frequency. We acknowledge that as a result of this, transient dips in response frequency are likely to be less pronounced, as they would occur relative to the entire range of SSD times. An interesting alternative approach to identifying dips in expected response frequencies in saccade stop signal tasks has been used elsewhere. Bompas et al. (2020) analysed saccade onset times locked to the stop signal rather than the target onset. This technique necessitates the creation of “surrogate” comparison distributions through random sampling of saccade onset times in the Go trial distribution.

Unlike Experiment 1, a significant effect of SSD on responding was found, but this took the form of an *increase* in saccade omissions at the longest SSD in younger children (Fig. 2B). This finding is also consistent with a transient inhibitory effect of the red flower signal, as at longer SSDs the effect would have less time to decay before the end of the response window. Omission errors were also found to be inversely correlated with age on Green flower trials, consistent with young children having more generalised difficulties in disengaging fixation from central stimuli.

As with Experiment 1, age influenced the variability of SRTs, with older children showing a reduced standard deviation compared with younger children. More precise timing of response intiation has been suggested as a strategy that participants’ learn to minimise commission and omission errors in stop signal tasks (Liddle et al., 2009), but as there was no instruction to countermand saccades in Experiment 2 this cannot explain the reduced variability in SRTs. Instead differences in variability likely reflect increased difficulty in disengaging fixation from central stimuli experienced by younger children in the task.

As all factors other than the instruction given to participants were closely matched between Experiment 1 and 2, differences and similarities in the results can be interpreted as reflecting the contribution of endogenous (cognitive) versus exogenous (simulus-driven) processes to performance. In the case of younger children, performance was very similar in the two experiments, indicating that the exogenous inhibitory effect of the stop signal explained much of their ability to “countermand” saccades in Experiment 1. Older children were able to overcome this influence to make saccades away from the Red flower in Experiment 2, whereas younger children continued to make response ommissions. With respect to the development of endogenous control therefore, comparison of the two experiments suggests that what develops most clearly with age in the countermanding task is not the ability to inhibit saccades, but the ability to overcome the exogenous inhibitory effect of central cues on saccade generation (i.e., oculomotor *disinhibition* rather than inhibition).

It should be noted that in both Experiment 1 and 2, the cartoon bee was redisplayed at fixation on “Stop” trials overlaid on top of the red flower (Fig. 1a). This was done because we were initially concerned that children would find countermanding reflexive saccades very difficult and so we wanted to make the stop signal as obvious as possible. This form of stop signal is also consistent with the classic implementation of the countermanding saccade task, where the reappearance of the fixation stimulus serves as the stop signal (Hanes & Carpenter, 1999). Given the findings of Experiment 2, we were concerned that this form of stop signal might be too visually salient and exert too strong a disruptive effect on saccade generation. It also seemed possible that the reappearance of the bee might have confused some children, as it meant that there were sometimes two bees on the screen at the same time. In order to check to what extent the findings were influenced by this methodological detail, we completed a final experiment in which the procedure was the same as Experiment 1 except that the bee did not overlap with the red flower at fixation (Fig. 1b). This time we also asked some of the children to complete a second block of trials to examine effects of practice and increased trial numbers in order to see if this might lead to more consistent effects of SSD on Stop trial errors, or evidence for learning strategies such as delaying or timing responses more precisely to reduce errors (Liddle et al., 2009).

## Experiment 3

### Methods

Thirty-seven children attending another year’s SSW event participated in Experiment 3, of which 33 met the inclusion criterion and completed at least 24 trials in each block with calibrated eye tracking of sufficient quality (two were excluded due to diagnosis of autism spectrum disorder). None of the children had completed the task at the previous SSW events. The participants included in the analysis ranged in age from 4.75 to 10.19, with a mean age of 8.09±1.84 and median age of 7.67 years. Fifteen of the participants also completed a second block of trials. For consistency and comparison purposes, ANOVA analyses of age were carried out using the same age classifications as in Experiments 1 and 2, though it should be noted that very few children in Experiment 3 fell into the youngest age category: 4 to 6 years (*N* = 4); 6 to 8 years: (*N* = 16); 8+ years (*N* = 13). The task instructions, timing, presentation and stimuli were identical to Experiment 1 apart from the deletion of the cartoon bee at fixation on Stop trials. The EyeLink 1000 system was also configured in chin rest mode for Experiment 3, rather than head free “sticker” mode as used in Experiments 1 and 2. This was done in order to minimize concurrent head movements.

### Results

#### Response errors

*Stop trials.* There was no main, *F*(2,32) = 1.471, *p* = 0.24, η_p_^2^ = 0.084, *BF*_*incl*_ = 0.588, or interaction effect, *F*(6,96) = 0.337, *p* = 0.916, η_p_^2^ = 0.021, *BF*_*incl*_ = 0.073, of categorical Age on commission errors. Stop trial errors were not significantly affected by SSD, *F*(6,96) = 1.770, *p* = 0.158, η_p_^2^ = 0.052, *BF*_*incl*_ = 0.672 although a nonsignificant positive trend was apparent in the data (Fig. 2C).

*Go trials.* Errors of omission on Go trials decreased significantly with categorical Age, *F*(2,35) = 16.377, *p* < 0.001, η_p_^2^ = 0.506, *BF*_*incl*_ = 671.672 (means 3–6 years = 44.7%, 6–8 years = 8.3%, 8+ years = 5.6%). This was also reflected in a significant negative correlation between omission errors and exact age, ρ = −0.528, *p* = 0.001.

#### Saccade reaction times

There was a significant main effect of Trial Type on mean SRTs, due to Stop trial errors having significantly shorter SRTs than Go trial responses (i.e., the opposite pattern to Experiments 1 and 2; mean Go = 383 ms versus Stop = 427 ms), *F*(1,32) = 5.49, *p* = 0.026, η_p_^2^ = 0.146, *BF*_*incl*_ = 14.420. There was a main effect of Age on SRTs, *F*(2,32) = 4.76, *p* = 0.015, η_p_^2^ *=* 0.229, *BF*_*incl*_ = 2.618, but no interaction between Age and Trial Type, *F*(2,32) = 0.281, *p* = 0.757, η_p_^2^ = 0.017, *BF*_*incl*_ = 0.277, although a stronger negative correlation was present between SRTs and exact age for Stop, ρ = −0.494, *p* = 0.003, relative to Go trials, ρ = −0.246, *p* = 0.153.

#### Estimated SSRT

Given that Go trial SRTs were faster on average than Stop trials and there was a trend towards higher commission errors at longer SSDs in Experiment 3, we applied the integration method (Logan & Cowan, 1984) to esimate individual participant SSRTs in the task. This method involves determining the point in the cumulative Go trial distribution at which the proportion of responses equals the overall rate of Stop trial errors and substracting the average SSD (93 ms) from this SRT value. This analysis gave an estimated SSRT of 157±49ms across all participants. There was no significant variation in SSRT with participant age as assessed through both correlations with monthly age, ρ = −0.141, *p* = 0.425, and an ANOVA against categorical Age, *F*(2,32) = 0.857, *p* = 0.434, η_p_^2^ = 0.052, *BF*_*incl*_ = 0.875 (means 3–6 years = 192±41ms; 6–8 years = 155±46 ms; 8+ years = 153±55).

Where omission errors are present on Go trials, it has been recommended to use the modified “integration with replacement” method to estimate SSRTs (Verbruggen et al., 2019). The involves recoding omissions as if they were correct Go trial responses with an SRT equivalent to the response window duration (1,000 ms) and ignoring the shortest SSD condition. This method produced longer estimates of SSRT, a significant negative correlation between SSRT and monthly age, ρ = −0.482, *p* = 0.003, and a significant effect of categorical Age, *F*(2,32) = 4.68, *p* = 0.017, *BF*_*incl*_ = 2.603 (means 3–6 years = 606±89 ms; 6–8 years = 372±43 ms; 8+ years = 297±48).

#### Saccade amplitude gain

A main effect of Trial Type was found on saccade amplitude gain, *F*(1,32) = 4.392, *p* = 0.044, η_p_^2^ *=* 0.121, *BF*_*incl*_ = 8.75 (means Stop = 0.888; Go = 0.911), with no main, *F*(2,32) = 1.306, *p* = 0.285, η_p_^2^ = 0.075, *BF*_*incl*_ = 0.700, or interaction effect, *F*(2,32) = 0.73, *p* = 0.490, η_p_^2^ = 0.044, *BF*_*incl*_ = 0.356 of Age. There was no significant effect of SSD on saccade amplitude, *F*(3,78) = 0.695, *p* = 0.558, η_p_^2^ = 0.026.

#### Dips in SRT distributions

**Fig. 8:**
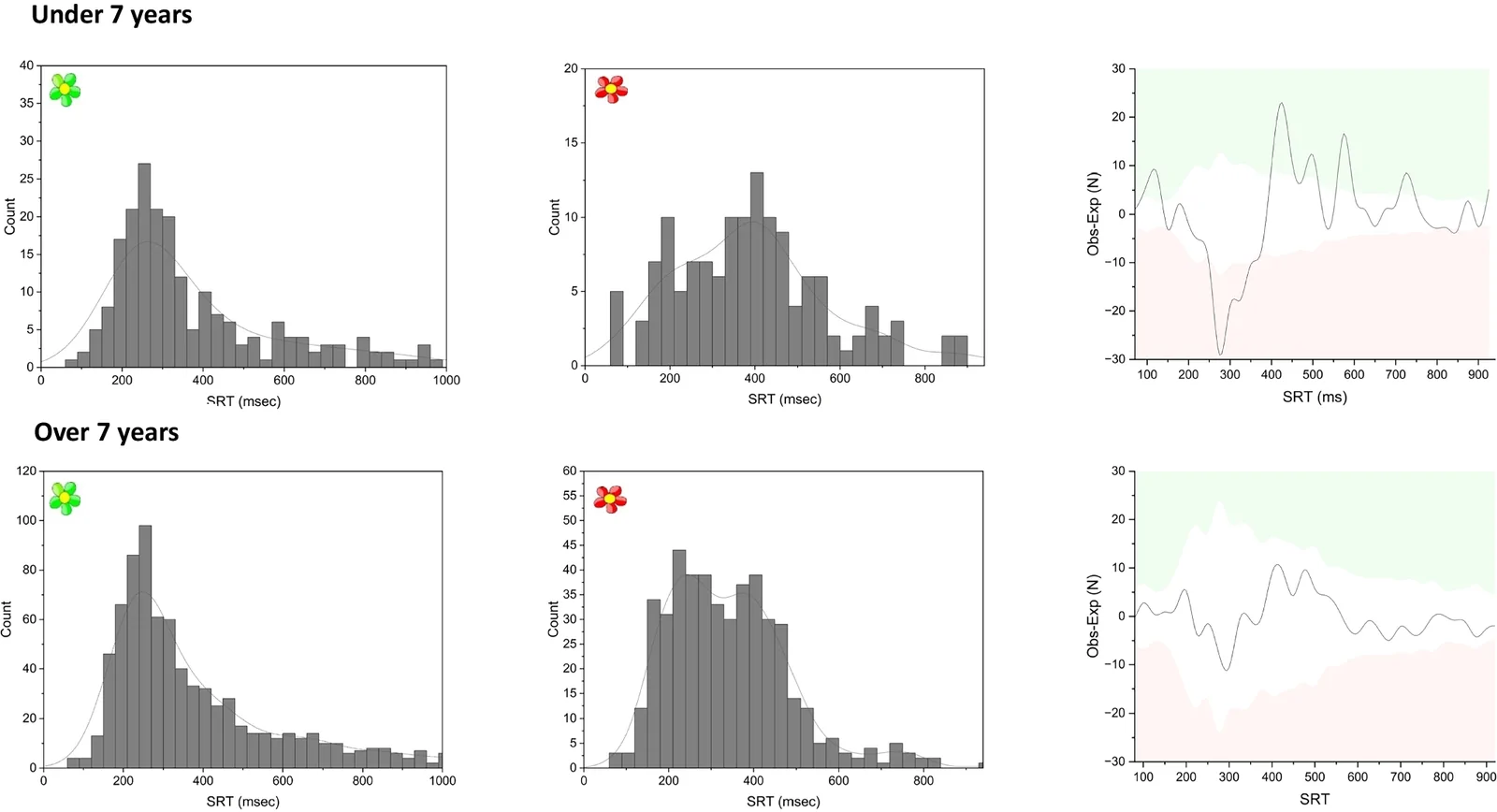
Target onset locked SRT distributions for correct Go trials and Stop trial errors in Experiment 3 (left panels) and observed versus expected values (right panels) for children younger or older than 7 years. Shaded regions show estimated critical values of the observed versus expected value difference

The same approach to analysing dips in the compound SRT distributions used in Experiment 2 was also applied in Experiment 3. Given the small number of participants in the 4–6-year age group, distributions were analysed for children Under or Over 7 years separately. Findings are shown in Fig. 8. For the younger children a significant dip was seen after the occurrence of the stop signal, but this was not observed in the older group (Fig. 8).

#### Practice effects

For the 15 children who completed two blocks, a two-way repeated-measures ANOVA was carried out, with Block (first versus second) and Error Type (omission versus commission) as factors. This showed an overall lower rate of errors in the second block (main effect of Block),,*F*(1,14) = 8.27, *p* = 0.012, η_p_^2^ = 0.371, *BF*_*incl*_ = 1.538, and higher rates of omission relative to commission errors (main effect of type), *F*(1,14) = 52.53,*p* < 0.001, η_p_^2^ = 0.79, *BF*_*incl*_ = 2.356. There was also a two-way interaction between Block and Error Type, reflecting a larger reduction in commission errors relative to omission errors in the second block, *F*(1,14) = 7.44, *p* = 0.016, η_p_^2^ = 0.347, *BF*_*incl*_ = 4.308 (Table 1). There was no effect of SSD on commission errors, *F*(3,42) = 1.195, *p* = 0.323, η_p_^2^ = 0.079, *BF*_*incl*_ = 0.564, and the effect of SSD on errors did not differ between the first and second block (interaction between SSD and block), *F*(3,42) = 1.23,*p* = 0.312, η_p_^2^ = 0.067, *BF*_*incl*_ = 0.229 (Fig. 2C).

**Table 1:** Mean error rates and saccade reaction times for Go and Stop trials in the first and second block of Experiment 3 (data from participants who completed both blocks only)

	Errors (%)		SRTs (ms)	
	Go	Stop	Go	Stop
Block 1	6.3±0.8	63.4±7.7	349±27	335±23
Block 2	4.6±1.4	50.0±6.9	411±27	339±13

The equivalent analysis of SRTs showed a main effect of Block, *F*(1,14) = 5.745, *p* = 0.031, η_p_^2^ = 0.084, *BF*_*incl*_ = 1.499, a significant main effect of Trial Type, *F*(1,14) = 5.689, *p* = 0.032, η_p_^2^ = 0.145, *BF*_*incl*_ = 2.325, as well as an interaction between Block and Trial Type, *F*(1,14) = 6.352, *p* = 0.024, η_p_^2^ = 0.066, *BF*_*incl*_ = 4.597. This was reflected in a selective increase in SRTs for Go trials in the second versus the first block of trials (Table 1), whereas Stop trial SRTs did not differ.

### Discussion

Experiment 3 differed from Experiment 1 only in the form of the Stop signal. In the first study, the cartoon bee was redisplayed on top of the red flower on Stop trials. This was in order to make the stop signal as clear as possible to children, as well as being consistent with the method used in the classic version of the countermanding task where reappearance of the fixation stimulus served as the stop signal (Hanes & Carpenter, 1999). By removing the overlapping bee we expected the stop signal to be less salient and for the exogenous inhibitory effect observed in Experiment 2 to be reduced. We were also concerned that the presence of two bees on the screen might have been confusing to children and may have made them more likely to maintain fixation on the central bee. The results of Experiment 3 were overall very similar to Experiment 1, suggesting that the presence of the overlapping bee at fixation could not explain the key findings. Omission and commission error rates were very close to those found in Experiment 1 and there was still no change in commission errors with age. Negative correlations were found between age and omission errors on Go trials, as well as well as with Stop trial error SRTs, as was the case in the previous studies.

**Fig. 9:**
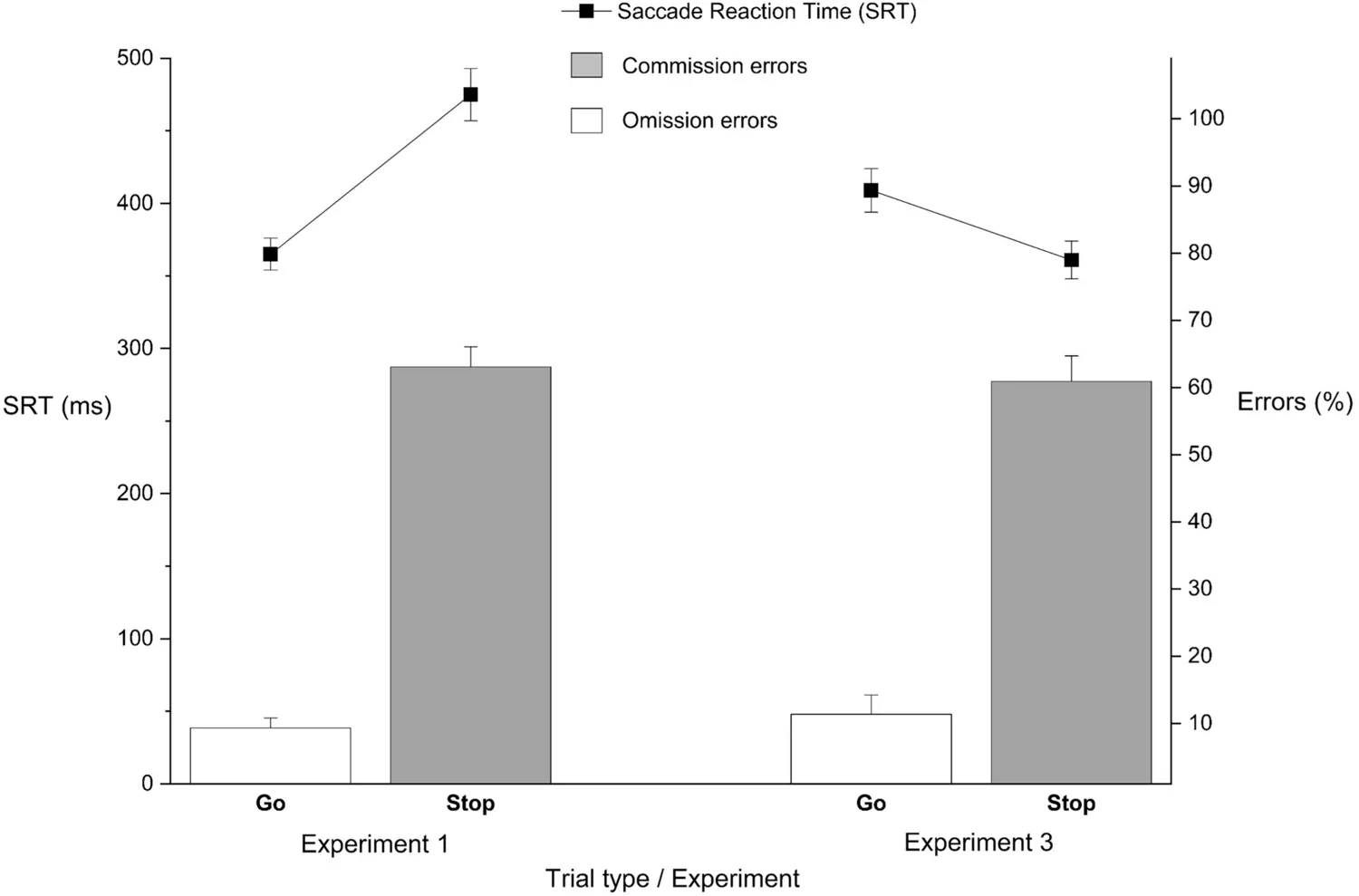
Errors and SRTs in Experiment 1 versus Experiment 3

One aspect of the findings that differed between Experiments 1 and 3 was the difference in mean SRTs between Go and Stop trial responses (Fig. 9). Whereas SRTs of Stop trial errors were significantly longer than Go Trials in Experiment 1, the reverse pattern was seen in Experiment 3. Superficially, this lends support to the classic horse-race model, as it suggests that the previous finding of longer SRTs on Stop trials was due to a methodological detail. However, even with the changed form of stop signal, dips were still apparent in Stop trial SRT distributions for younger children (Fig. 8). This finding is novel compared to previous studies as it shows that a change in colour (rather than the reappearance) of the fixation stimulus can cause a transient inhibitory effect on saccades. It is also problematic for accounts that assume contextual equivalence between Go and Stop trials (other than the demand to cancel a response). Instead, the cognitive and perceptual demands of stop trials are influenced by the characteristics of the stop signal. Sensitivity to these demands could conceivably vary across particular participant groups and with individual traits in addition to age, reducing the validity of the countermanding task as a straighforward test of inhibitory control.

As Stop trial error SRTs were not significantly longer than Go trial SRTs in Experiment 3, SSRTs were estimated using the integration approach (Logan & Cowan, 1984). Using the standard integration method, no significant variation in SSRT was found across age groups, but when applying the integration with replacement method (Verbruggen et al., 2019), SSRT was found to reduce with increasing age. This is consistent with a reduction in the average time to threshold of the internal stop process with age, as has been found for manual stop signal tasks (Williams et al., 1999). However, as the integration with replacement method treats omission errors as if they were long SRT responses, the significant effect of age on saccade omissions represents a complicating factor in interpretting the analysis, as more hypothetical 1,000 ms values were inserted into younger children’s Go trial SRT distributions. This is a concern as younger children have higher rates of omissions even when there is no instruction to countermand (Experiment 2). Given this, and the established exogenous inhibitory effect of the stop signal, some caution should be taken in concluding that the effect of age on SSRT directly reflects maturation of an internal stop process.

A secondary aim of Experiment 3 was to examine whether given more practice, children could improve their performance and produce more consistent effects of SSD. Errors were found to be significantly reduced in the second block of trials, with a greater reduction for commission relative to omission errors. Go trial SRTs were also significantly longer in the second block, consistent with strategic delaying of responses to minimise errors (Table 1). A positive trend in commission errors against SSD was apparent (Fig. 2C), although this was flatter than would be predicted by the classic horse-race model, which would produce very low commission errors at short SOAs and close to 100% errors at long SSDs. Flat SSD functions are suggestive of the influence of a random process on performance independent of SSD, whereby sometimes the stop signal is processed and enacted upon and at other times it is not. A process of this type is included in existing computational models as a failure or inattention parameter (Bompas et al., 2020; Salinas & Stanford, 2013), with a fixed probability of the stop process not being triggered irrespective of SSD. The current results suggest that this inattention parameter may exert a more dominant influence on children’s performance in the task than the stop process itself.

## General discussion

A general issue faced by researchers working with young children is the extent to which age-related changes in performance truly reflect development of cognitive, perceptual, and motor processes, or simply arise due to children not understanding task instructions. We approached this issue by presenting the countermanding saccade task as a game, using simple child-friendly instructions displayed on the screen, which were simultaneously read out aloud by the researcher at the start of the test. Reminder prompts were also repeated prior to the start of each test block (e.g., “remember if the flower turns red look straight ahead instead”). In fact, a notable feature of our findings was how frequently children did not saccade to the target on stop trials. Three-to-6-year-old children withheld a response on 44% of Stop trials alongside 12% omission errors in Experiment 1. As well as being surprising relative to our prior expectations, this suggests that even the youngest children understood the task at some level.

Although no effect of age was seen for Stop trial errors, negative correlations were found between age and response omissions across experiments, including in the uninstructed version of the task used in Experiment 2. Again, it might be argued that response omissions occurred because younger children did not understand the instructions. This was a particular concern as the cartoon bee was redisplayed at fixation along with the red flower on Stop trials in Experiments 1 and 2, so some children might have just decided to stay looking at the bee in the centre of the screen rather than looking towards the bee on the left or right. However, this cannot explain the transient dips in SRT distributions associated with the stop signal onset in younger children. These were found in the SRT distributions for younger children even when the overlapping bee had been deleted (Experiment 3). Errors of omission were also increased in younger children on Green flower/Go trials, such that they are unlikely to reflect comprehension difficulties or confusion about the bee displayed at fixation.

Instead of reflecting failures to understand task instructions, it is suggested that saccade omissions, as well as dips in SRT distributions and long SRTs on Stop trials, reflect a generalised difficulty experienced by younger children in disengaging ocular fixation from salient central cues. This is consistent with our observations of children performing eye movement tasks over a 12-year period at the SSW event, who often show a “stickiness” in fixation control that can sometimes make eye tracking setup and calibration challenging.^1^ This feature of developing oculomotor control can lead to younger children being deemed unsuitable participants for eye movement research and might be misinterpreted as failure to comply with very simple instructions (i.e., “follow the bee”). More significantly, immature fixation control could lead to an underestimation of children’s cognitive abilities in tasks that either implicitly or explicitly require fixational eye movements. For example, previous work has shown that a superficial adjustment to experimental procedures in order to facilitate fixation disengagement (removing overlapping central fixation cues), lead to more equivalent performance between younger and older children in a social attention task (Gregory et al., 2016). Conversely, sticky fixation control could lead to an overestimation of younger children’s abilities when a task requires them to maintain attention in the face of distracting peripheral stimuli (as is the case with the countermanding saccade task).

The sticky fixation effect in children may be related to previously reported “obligatory attention”: an inability to look away from objects at fixation in babies younger than 12 months (Johnson et al., 1991; Stechler & Latz, 1966). It has been suggested that this reflects excessive tonic cortical inhibition of the mid-brain superior colliculus (SC) via the substantia nigra pars reticulata (SNPR) early in infant development, which progressively dissipates as these top-down pathways mature (Johnson et al., 1990). The SC itself comprises a rostral zone within which neuronal activity is associated with sustained fixation, whilst neurons in caudal regions are organised into a spatiotopic map of peripheral target locations and saccade vectors (Munoz & Wurtz, 1992, 1993). Interestingly, these same neural pathways have been implicated in the performance of the countermanding saccade task (Hanes et al., 1998). Cortical regions, including the frontal eye fields, are thought to provide spatially mapped input onto the SC to control the initiation of voluntary saccades, whilst disinhibition of the SNPR is required to actively disengage from the currently foveated stimulus. One possibility suggested by the current findings is that the cortical basal ganglia pathway responsible for disengaging fixation continues to develop beyond the age of 12 months through early childhood, explaining the larger inhibitory effect of stop signals and increased omission errors in children performing the countermanding task.

Future studies of countermanding saccades in children might overcome complications arising from immature fixation control using an auditory stimulus as the stop signal. We anticipate that auditory stop signals would produce clearer effects of age on errors as they would not directly influence engagement of fixation and saccade initiation. However, previous studies suggest that auditory stop signals might enhance stochastic influences on stopping, with such signals being more likely to be missed or ignored (Cabel et al., 2000; Salinas & Stanford, 2013). As such It would still not be clear whether any effect of age in an auditory countermanding task reflected processes of inhibitory control, sustained attention or attentional load, especially as such a task would also necessitate dual attention to visual and auditory modalities. Another modification to the classic countermanding task which could be investigated would be to use a wider range of SSDs, including negative SSDs (i.e., stop signal prior to target onset) and longer SSDs. Bissett et al. (2021) have suggested that longer SSDs might negate the transient disruptive effects of the stop signal on target processing by temporally distancing the stop signal from the target onset. The same authors point out that a problem with longer SSDs is that they may incentivise strategic delaying of responding, leading to more omission errors. Negative SSDs are also problematic as they undermine the nature of the countermanding task as a test of reactive inhibition, as participants are effectively being asked to cancel a response programme before it has been initiated (Bissett et al., 2021).

Although SSRTs were found to reduce with age in Experiment 3 (when estimated using the integration with replacement method), individual children’s error rates rarely showed a clear positive linear relationship with SSD such that it was not possible to estimate SSRTs directly from SSD error functions. In order to do so, it would likely be necessary to adjust the range of SSD values against each child’s performance via a staircase optimisation procedure and require much longer practice and test blocks. This approach was not considered feasible as long test blocks would not be tolerated by children. Instead, we choose a fixed range of SSDs based on those used in other published studies (Hanes & Carpenter, 1999; Hanisch et al., 2006). We also subsequently tested a small group of eight adult controls using the same child-friendly countermanding saccade task to see if consistent effects of SSD were found. Participants included a mixture of individuals who were naïve with respect to the task (undergraduate students) and those who were experienced oculomotor researchers with knowledge of the countermanding task and horse-race models (two of the co-authors and a post graduate student). Each participant completed three blocks using either the method of Experiment 1 or 3. Although mean SRTs were shorter in adults relative to children (Go trial mean SRT adults = 326 ms; Children in Experiments 1 & 3 combined = 383 ms), the findings with respect to commission errors and SSD were very similar. There was no consistent effect of SSD on commission errors across the adult participants, with only three of the participants showing a positive trend in errors with increasing SSD (Supplementary figure). Performance also did not appear to be clearly influenced by the exact procedure used or the experience of the participants. We acknowledge that introducing SSD values closer to the end of the response window would likely lead to commission errors rates consistently close to 100%, but errors at the shortest SSD were also higher than would be predicted by a simple race model, with the majority of adult participants showing error rates over 40%. This suggests that the lack of a consistent effect of SSD is not unique to children and that saccade countermanding is also subject to stochastic influences in adults, at least when using the low trial numbers and fixed SSDs necessitated by working with children. A plausible candidate for this stochastic process is the failure/inattention parameter in existing computational models. This assumes that the stop signal is not processed on a fixed proportion of trials independent of SSD, leading to a failure to initiate a stop process (Bompas et al., 2020; Salinas & Stanford, 2013), with the rate of commission errors at zero SSD proprosed as an indicative baseline frequency for such trigger failures (Verbruggen et al., 2019).

A question of practical importance arising from this study is whether the countermanding task is a useful test for assessing typical and atypical development of inhibitory oculomotor control. We conclude that whilst the task highlights a very interesting feature of children’s developing oculomotor control (difficulty with fixation disengagement), it should not be treated as a straightforward test of inhibition. No significant effects of age were found on stopping ability, whereas the other widely used test of oculomotor inhibitory control (the anti saccade task) produces consistent and replicable age effects with similarly short testing blocks (e.g., Bucci & Seassau, 2012; Hodgson et al., 2025; Sumner et al., 2018). Whilst the anti saccade task clearly places demands on processes other than response inhibition (e.g., sensorimotor transformation and voluntary saccade programming), the finding that stop signals have an exogenous inhibitory effect in children also precludes any straightforward interpretation of the countermanding task as a test of endogenous inhibitory control. Adaptations to the task procedure for children might address some these issues (see above), but their effectiveness cannot be determined without further detailed study.

## Summary and conclusions

Our findings in children contribute to an accumulating body of evidence that challenges the idea that countermanding tasks primarily index higher level cognitive control (Bompas et al., 2020). Stop signals presented at fixation were found to exert a strong exogenous inhibitory effect on saccade generation in children and this effect correlated inversely with age, contributing to high rates of response omissions in younger children even when no instruction was given to countermand saccades. A consistent lack of variation of commission errors with SSD suggests horse-race models may not be the best framework for understanding children’s performance in the task. Instead, the findings are consistent with the influence of a stochastic process independent of SSD, whereby inattention to the stop signal leads to failures to initiate a stop process (trigger failures). Overall, it is concluded that whilst the countermanding task sheds light on aspects of children’s eye movement and fixation control (“sticky” fixation), care should be taken in interpreting it as a pure test of endogenous inhibition. Instead, simpler tasks such as the anti-saccade (Hallet & Adams, 1980), gap-overlap (Saslow, 1967), and remote distractor tasks (Walker et al., 1997; Zhang et al., 2020) might prove more useful for assessing children’s developing oculomotor control.

## Supplementary Information

Below is the link to the electronic supplementary material.

Supplementary file1 (XLSX 15 KB)

Supplementary file2 (PDF 228 KB)

## Data Availability

All data and materials described in the manuscript are available from the corresponding author on request.

## Footnotes

1 The phenomenon does not appear to be strongly affected by target eccentricity as we have observed it in tasks with eccentricities of ±9° (Gregory et al., 2016) as well as with the larger values (±15°) used here.

## Code availability

All experiment code, analysis scripts and spreadsheets are available on request and via Open Science Forum: https://osf.io/q4pgh
