# Supplementary material for: Countermanding saccades in children
Source: Atten Percept Psychophys. 2026 Oct 29;88(8):217. doi: 10.3758/s13414-026-03342-4 (PMC13723689; doi:10.3758/s13414-026-03342-4)
Supplement: Supplementary file 1 [file 13414_2026_3342_MOESM2_ESM.pdf]

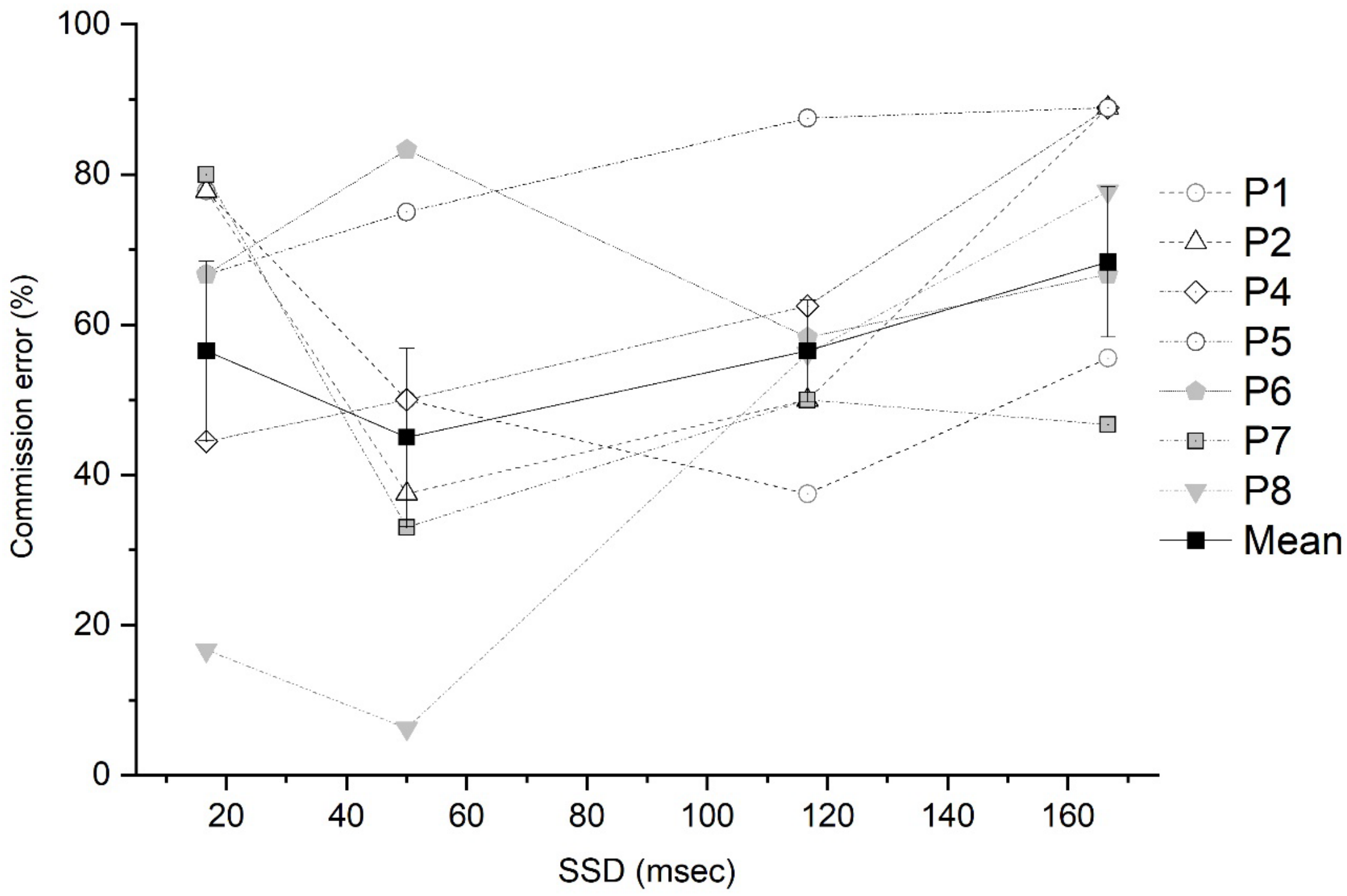


**Supplementary Figure:** Adult participant's performance in the children's countermanding saccade task showing relationship between commission errors and stop signal delay (SSD). All participant completed 3 blocks of the task according to the trial procedure of Experiment 1 (P1-5) or Experiment 3 (P6-8). Open symbols were undergraduate students with no knowledge of the countermanding task and underlying theory. Filled symbols were participants who had detailed knowledge of the task and theoretical models of inhibitory control (including 2 of the co-authors).
